# Rapid sintering of silicon nitride foams decorated with one-dimensional nanostructures by intense thermal radiation

**DOI:** 10.1088/1468-6996/15/4/045003

**Published:** 2014-07-08

**Authors:** Duan Li, Elisângela Guzi de Moraes, Peng Guo, Ji Zou, Junzhan Zhang, Paolo Colombo, Zhijian Shen

**Affiliations:** 1 Department of Materials and Environmental Chemistry, Arrhenius Laboratory, Stockholm University, S-106 91 Stockholm, Sweden; 2 Berzelii Center EXSELENT on Porous Materials, Stockholm University, S-106 91 Stockholm, Sweden; 3 Dipartimento di Ingegneria Industriale, University of Padova, Via Marzolo 9, I-35131 Padova, Italy; 4 College of Materials and Mineral Resources, Xi’an University of Architecture and Technology, 710055 Xi’an, People’s Republic of China; 5 Department of Materials Science and Engineering, Pennsylvania State University, University Park, PA 16801, USA

**Keywords:** silicon nitride, foam, spark plasma sintering, nanowire, thermodynamics

## Abstract

Silicon nitride foams were prepared by direct foaming and subsequent rapid sintering at 1600 °C. The intense thermal radiation generated under the pressureless spark plasma sintering condition facilitated necking of Si_3_N_4_ grains. The prepared foams possessed a porosity of ∼80 vol% and a compressive strength of ∼10 MPa, which required only ∼30 min for the entire sintering processes. Rapid growth of one-dimensional SiC nanowires from the cell walls was also observed. Thermodynamic calculations indicated that the vapor–liquid–solid model is applicable to the formation of SiC nanowires under vacuum.

## 1. Introduction

Silicon nitride ceramic foams are attractive materials for thermal/electrical insulators and hot gases filters due to their relatively high strength, good thermal stability, superior wear and corrosion resistance [1, 2]. This family of porous materials can be prepared by partial sintering or various foaming methods (e.g. direct foaming followed by setting (by particle stabilization or gel-casting), the replication of a polymeric template, the pyrolysis of porous preceramic polymers, freeze-casting, the use of sacrificial templates) followed by sintering [3–6]. Sintering is an essential processing step for gaining mechanical stability and rigidity of the ceramic foams. Sintering of silicon nitride often encounters a difficulty related to its easy disassociation at the high temperatures necessary for sintering [7, 8]. This difficulty appears more critical when conventional pressureless sintering techniques are of concern, because they require elevated temperatures (> 1800 °C) and prolonged dwelling time (a few hours) for sufficient densification to occur [9–11]. In order to overcome this difficulty, advanced sintering techniques such as spark plasma sintering (SPS) [12, 13] and microwave sintering [14, 15], have been employed to sinter the silicon nitride family of ceramics. They are able to ensure densification at lower temperatures with shorter dwelling times [16, 17]. In addition, by applying these novel processing concepts microstructures with unique features were obtained [12, 13, 18].

In this study, we modified the SPS set-up to allow pressureless sintering of Si_3_N_4_ green foams prepared by a direct foaming method based on surfactant stabilization. Rapid sintering was realized by intense thermal radiation. Rigid Si_3_N_4_ foams decorated with SiC nanowires (NWs) grown on the cell walls were obtained. The mechanism of rapid sintering and the mechanical strength of the foams were investigated. In addition, the formation mechanism of such 1D nanostructures was studied based on thermodynamic calculations.

## 2. Experimental procedures

**Figure 1.**
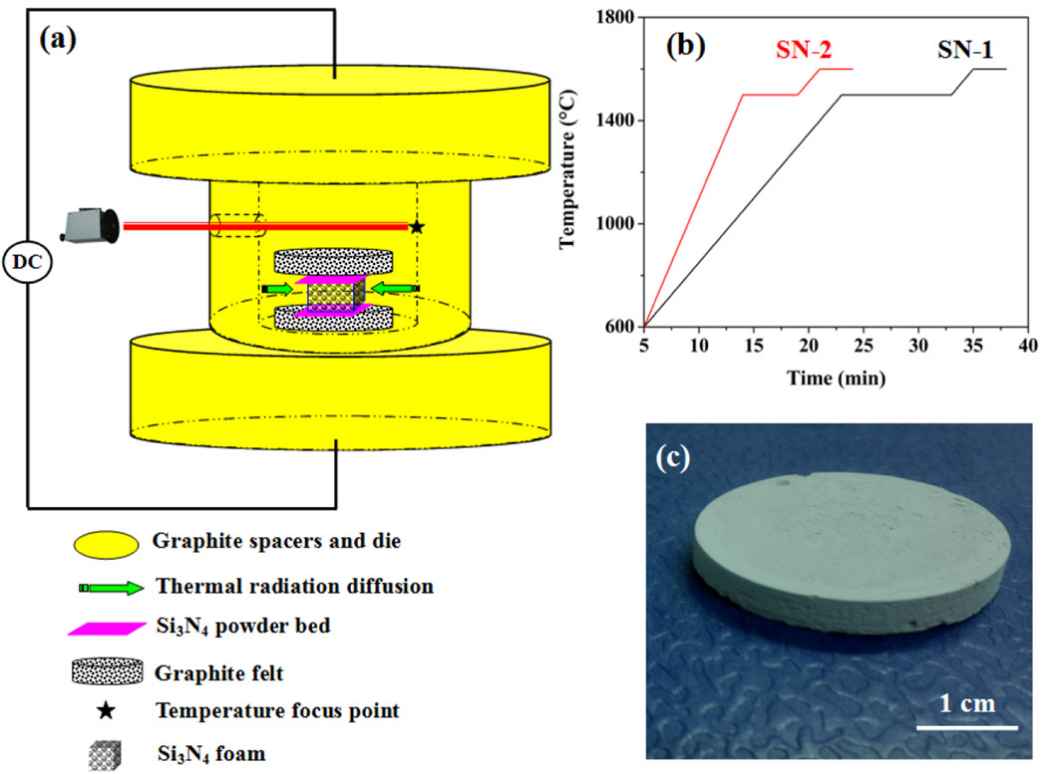
Graphic description of the ITRS process: (a) schematic diagram for the modified SPS set-up inside the chamber; (b) sintering regimes for samples SN-1 and SN-2; (c) optical image of the as-sintered SN-1 foam after gentle polishing.

We used Si_3_N_4_ powder (grain size *d*_50_ = 0.6 *μ*m, purity ⩾ 96 wt%, main impurity was trace Fe, *α* phase ⩾ 91.5%, Yantai Tomley Hi-tech New Materials, Yantai, Shandong, China) with the sintering additives of 5 wt% Y_2_O_3_ (grain size *d*_50_ = 50 nm, purity ⩾ 99.95 wt%, Inframat Advanced Materials L.L.C., Manchester, New Hampshire, USA) and 5 wt% MgO (particle size *d*_50_ = 4.6 *μ*m, purity ⩾ 99.99 wt%, Bitossi Ceramiche S.R.L., Montelupo Fiorentino, Firenze, Italy) for preparation. These starting powders were mixed and ball milled in ethanol for 4 h. The obtained slurry was then dried and sieved through a 300 *μ*m screen, followed by heat treatment in air at 600 °C for 2 h, in order to form a thin oxide layer on the particle surfaces. The powder mixture was then added stepwise to deionized water containing 1 wt% (relative to the powder mixture) dispersing agent polyacrylic acid (PAA, Sigma-Aldrich Sweden AB, Stockholm, Sweden) under constant stirring. Subsequently, the Si_3_N_4_ slurry containing 35 vol% of solids was homogenized and dispersed by ball milling for 2 h. The emulsification was induced by adding 50 vol% octane (C_8_H_18_, Sigma-Aldrich Sweden AB, Stockholm, Sweden) and 0.22 vol% (with regard to the suspension) nonionic surfactant polysorbate Tween^@^ 80 (VWR International, Bedfordshire, UK) with stirring at 700 rpm for 3 min. Afterwards, in such a surfactant-stabilized emulsified suspension, foaming was provided by octane droplet evaporation in ambient air for 24 h. The evaporation of the oil (alkane) phase and concurrent drying (water evaporation) enabled the transition of the emulsified suspensions into green solid foams. Different solid loadings, oil contents and stirring speeds were tested, and the above-mentioned foaming process is based on the optimized procedure. Rapid pressureless sintering was conducted in a modified SPS set-up (Dr Sinter 2050, Sumitomo Coal Mining, Tokyo, Japan) under vacuum (see figure 1(a)). The green foams were loaded in a covered cylindrical graphite crucible with an inner diameter of 50 mm and outer diameter of 70 mm. The samples were protected by a Si_3_N_4_ powder bed and isolated by graphite felts. Two sintering regimes, namely SN-1 and SN-2, were set as follows (see figure 1(b)): the temperature was automatically raised to 600 °C over a period of 5 min, and from there onwards it was monitored and regulated by an optical pyrometer focused on the wall centrally inside the crucible through a hole of ∼5 mm in diameter. For SN-1, the sample was heated to 1500 °C with a heating rate of 50 °C min^−1^ and maintained for 10 min, while the heating rate and dwell time were 100 °C min^−1^ and 5 min for SN-2. All samples were then heated to 1600 °C at 50 °C min^−1^ and held for 3 min at that temperature. Rigid Si_3_N_4_ foam disks with a diameter of 30 mm and a height of 8 mm were obtained (see figure 1(c)). Samples were labeled according to the sintering regime adopted.

Crystalline phase analysis was performed using a PANalytical X’pert PRO MPD diffractometer (PANalytical, Almelo, Netherlands) using CuK*α* radiation (*λ* = 1.5418 Å) at room temperature. The weight fractions of the *α*- and *β*-Si_3_N_4_ were evaluated by the direct comparison method [19]. A micro-Raman spectrometer (LabRAM HR 800) with a spectral resolution of 0.5 cm^−1^ was employed for phase identification by using a Nd:YAG laser (532 nm, 50 mW). Before the measurement, the apparatus was spectrally calibrated using the 520.7 cm^−1^ Raman line from a silicon wafer. The porosity values of the samples with pore diameter ranging from 100 nm to 100 *μ*m were determined using mercury intrusion porosimetry (Micromeritics AutoPore III 9410, Norcross, Georgia, USA). The surface tension and the contact angle of the Mercury were set to 0.485 N m^−1^ and 130°, respectively. The microstructure of the foams was characterized by a field emission scanning electron microscope (FE-SEM, JSM-7000F, JEOL, Tokyo, Japan) and a Schottky-type field emission transmission electron microscope (TEM, JEM-2100F, JEOL, Tokyo, Japan) operated at 200 kV, equipped with an energy-dispersive x-ray spectroscopy (EDX) detector. Electron diffraction (ED) data were collected on a LaB_6_ based transmission electron microscope (TEM, JEM-2100, JEOL Ltd, Tokyo, Japan) at 200 kV. For TEM study, the sample was crushed into powder and dispersed in ethanol. The sizes of grain, cell and cell window were measured using the linear intercept method based on the SEM images (ASTM E0112-10). The compressive strength of the foams was measured at 25 °C using a universal testing machine (1121 UTM, Instron, Norwood, MA, USA). The cross-head speed was 1.0 mm min^−1^ and the compressive load cell was 5000 N. A minimum of five specimens with a nominal dimension of 10 × 10 × 10 mm^3^ were tested per data point (ASTM C133-97). Thermodynamics during the formation process (25 ∼ 1600 °C) was investigated by a commercial software (HSC Chemistry for Windows 6.1, Outokumpu Research Oy., Pori, Finland), assuming that the total gas pressure was 10 Pa at each equilibrium condition.

## 3. Results and discussion

### 3.1. Sintering by intense thermal radiation

**Figure 2.**
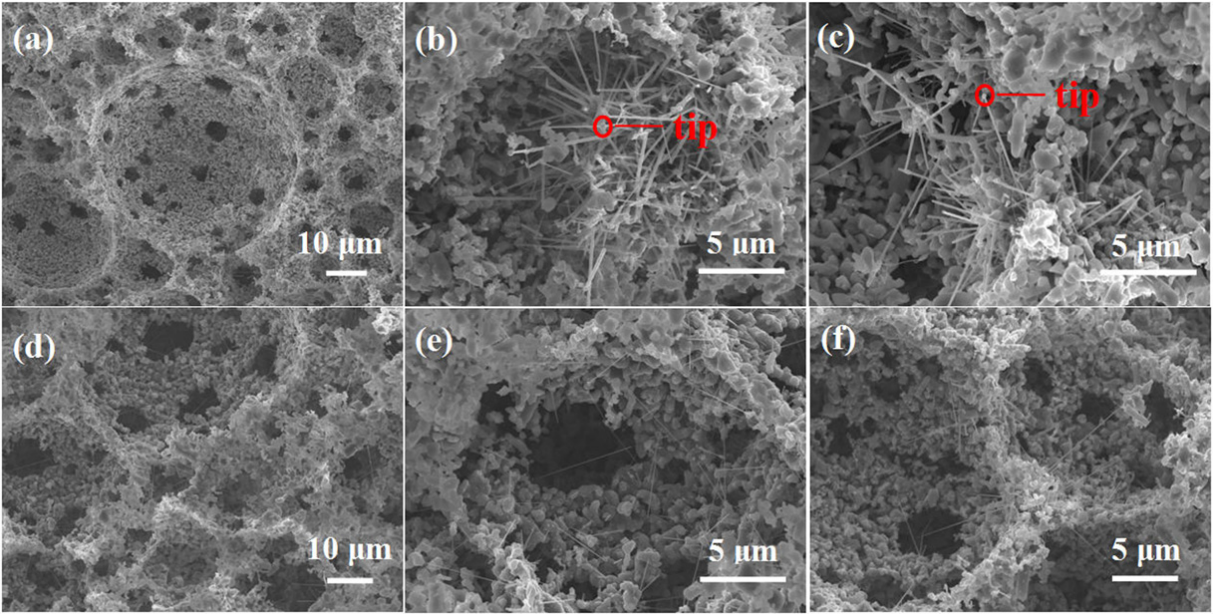
SEM images of the as-sintered SN-1 (a), (b) and (c) and SN-2 (d), (e) and (f) samples showing (a) and (d) a macroporous structure with cells, windows and cell walls; (b) (c) (e) and (f) growth of NWs from the cell walls. Images (b) and (c) clearly reveal a tip-body structure of the NWs.

The microstructures of the as-sintered Si_3_N_4_ foams are shown in figure 2. For both SN-1 and SN-2 samples, spherical cells interconnected through several windows and surrounded by walls can be observed in figures 2(a) and (d). SN-1 and SN-2 samples possessed porosity values of 79.4 ± 1.0 vol% and 79.3 ± 1.0 vol%, respectively (see table 1). The average cell size for samples SN-1 and SN-2 was comparable (25.0 ± 2.2 *μ*m and 27.0 ± 1.7 *μ*m, respectively), and both samples had a similar cell window size (6.0 ± 0.4 *μ*m and 7.0 ± 0.4 *μ*m, respectively). Several NWs formed when samples were processed according to both sintering regimes, and appeared protruding from the cell walls, as can be seen in figures 2(b), (c), (e) and (f) (see later). Apart from the foaming process, sintering contributed significantly to the development of all the above-mentioned microstructural features. Sintering of silicon nitride is a complicated process involving atomic diffusions, densification/void elimination, phase transformation, decomposition and grain coarsening/growth [8]. Based on our previous investigations, the sintering temperature of Si_3_N_4_ used in our study was optimized to be 1600 °C, below which no densification occurred and above which too strong disassociation would appear.

**Table 1.** Properties of the as-sintered Si_3_N_4_ foams.

	^a^Average pore size (*μ*m)				
Sample	Cell	Window	^b^Porosity (%)	^c^ *β*-Si_3_N_4_ phase content (*β*/(*α* + *β*) wt%)	^a^Average grain size (*μ*m)
Starting powder mixture	—	—	—	5	0.6
SN-1	25.0 ± 2.2	6.0 ± 0.4	79.4 ± 1.0	30	2.0
SN-2	27.0 ± 1.7	7.0 ± 0.4	79.3 ± 1.0	10	1.2

a Calculated by the numerical interpolation method from the SEM images by using an interpolation factor of 1.5. The average grain size before sintering was measured to be ∼0.8 *μ*m.

b Calculated by assuming that the theoretical density of 100% dense Si_3_N_4_ ceramic is 3.4 g cm^−3^.

c Determined from the intensities *I*_*α*_ and *I*_*β*_ of the (2 0 1) *α*-Si_3_N_4_ and (1 0 1) *β*-Si_3_N_4_ powder x-ray diffraction (PXRD) peaks, respectively, by the direct comparison method. The following expression was used to determine fraction *x*_*β*_ of the *β*-Si_3_N_4_ phase:

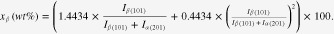

Sintering is facilitated by heating, and there are three ways of heat transport, namely, thermal conduction through contact, thermal convection by fluids, and radiation via an irradiator [20, 21]. At low temperatures, conduction and convection are crucial while thermal radiation dominates at elevated temperatures [20, 21]. During conventional sintering processes, all these mechanisms are simultaneously active. In the modified SPS set-up used in this study the contribution of radiative transfer to the samples from the surrounding hot wall of the graphite crucible was significant because: (i) no direct contact occurred between the crucible and the samples, so direct conduction could be excluded; (ii) a vacuum atmosphere (∼10 Pa) was used to suppress convective transport; (iii) sintering was conducted at a high temperature of 1600 °C at which thermal radiation was encouraged; and (iv) a heating volume of *Φ*50 mm × 40 mm for the sample disks with size of *Φ*30 mm × 8 mm enabled high-intensity thermal radiation. All these factors made the heating process effective and homogeneous. In the discussion below this sintering strategy will be referred to as sintering by intense thermal radiation (SITR).

### 3.2. Neck formation and mechanical properties

**Figure 3.**
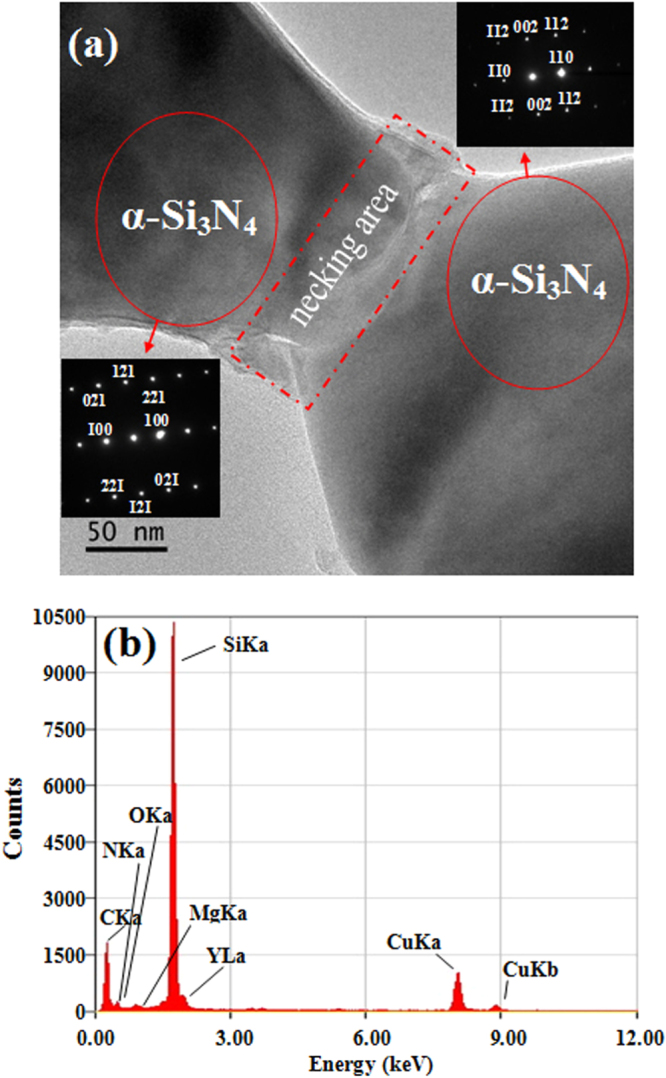
TEM/SAED/EDX analyses of sample SN-1. (a) The TEM image shows the necking area between two Si_3_N_4_ grains, and the indexed SAED patterns indicate that the phase of the two grains is *α*-Si_3_N_4_; (b) the EDX result of the necking area illustrates the chemical composition.

The TEM analysis in figure 3 shows the formation of a neck between two Si_3_N_4_ grains in sample SN-1 after sintering at 1600 °C. The indexed selected-area electron diffraction (SAED) patterns indicate a clear necking area between two *α*-Si_3_N_4_ grains. The EDX result shows the presence of Si, Y, Mg, O, and N (all the above could be from the liquid phase), as well as Cu (from the Cu grid) and C (from the carbon film). The PXRD data, summarized in table 1, illustrate that the *α* → *β* transformation occurred (starting from 1500 °C) and was enhanced by increasing the dwell time, since the SN-1 sample which encountered a longer dwell time at 1500 °C had a higher *β* phase content (30 wt%) than sample SN-2 (10 wt%). The average grain size coarsened by a factor of ∼3 (SN-1) or ∼2 (SN-2) times with respect to the starting powder (see table 1). Extensive studies have shown that the reconstructive nature of the *α* → *β* transformation is realized by the solution–re-precipitation mechanism [7, 8, 11, 13, 22]. The equiaxed *α*-Si_3_N_4_ grains were metastable during sintering and transformed irreversibly to elongated *β*-Si_3_N_4_ grains, which formed *in situ* through a solution–precipitation process in the presence of a liquid phase containing silicon oxynitride [11]. The existence of the oxynitride liquid phase can be confirmed by the EDX result.

**Figure 4.**
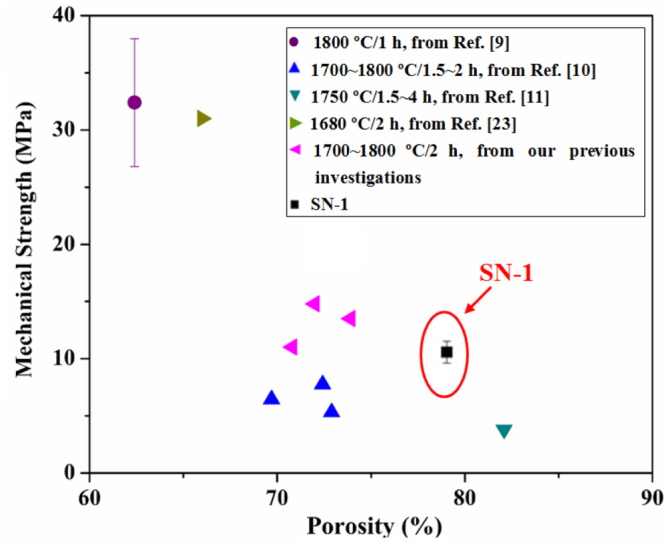
Mechanical strength of the as-sintered sample SN-1 as a function of the porosity, compared with those prepared by conventional sintering at different sintering temperatures and dwell times. The data were collected from literature and our previous investigations.

The effective sintering and necking led to the development of mechanical strength in the foams. Figure 4 compares the compressive strength in the SN-1 sample and other highly porous Si_3_N_4_ ceramics reported in the literature [9–11, 23]. These foams were prepared by conventional sintering at >1600 °C with dwelling time being hours, and the mechanical strength was measured in terms of flexural or compressive strength. It is well known that the mechanical strength of porous materials greatly decreases with increasing porosity [24, 25]. With a total porosity of ∼80 vol%, the SN-1 sample possessed a strength of 9.9 ± 0.9 MPa. The value is even comparable to the samples with lower porosity values (∼70 vol%), revealing that the SITR process is effective and efficient given that only ∼30 min for the entire sintering processes were needed. Such strengthening effect of necking has also been observed during pulse electric current sintering of porous alumina [26, 27].

### 3.3. Formation of 1D nanostructures

**Figure 5.**
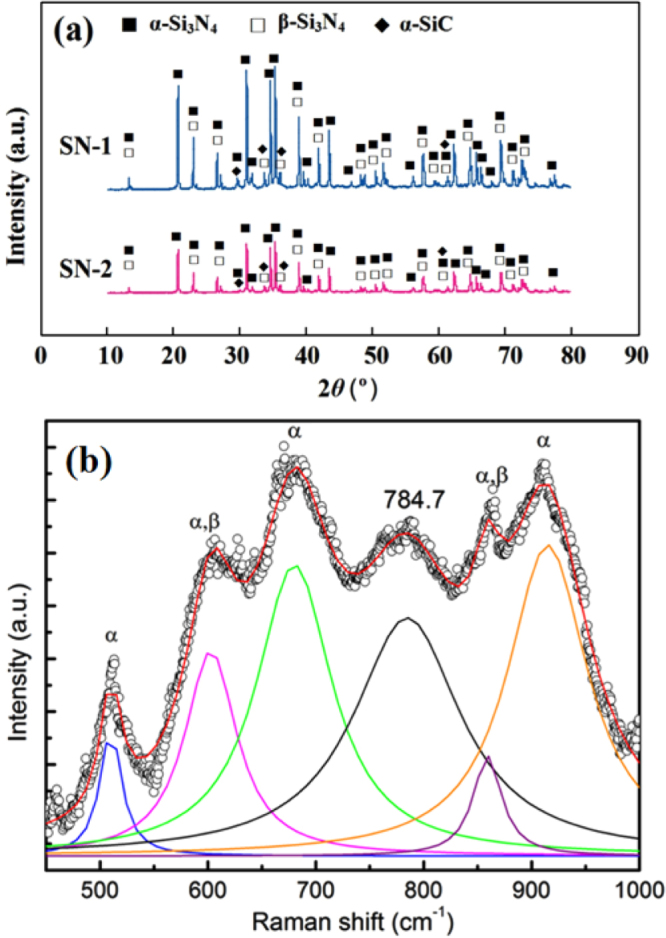
(a) PXRD patterns of the sintered samples SN-1 and SN-2. (b) Raman spectrum of sample SN-1. The broadening of the Raman peak at 784.7 cm^−1^ is due to the disordered structure of SiC.

**Figure 6.**
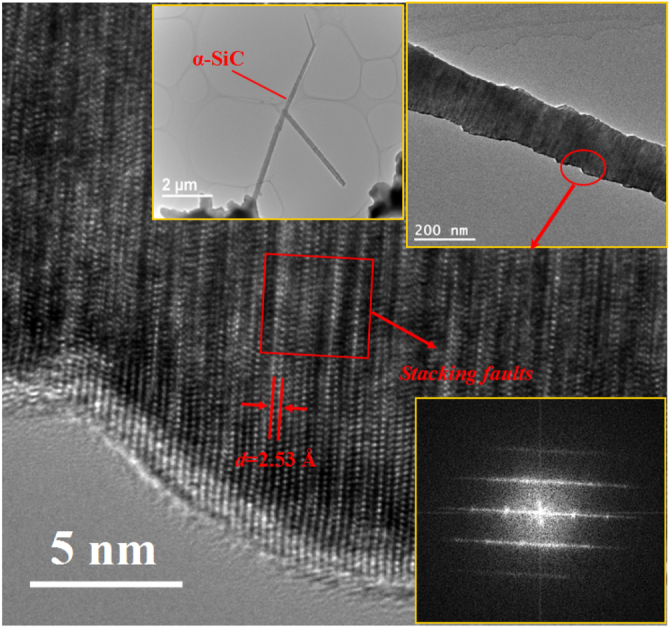
TEM analyses of the NWs from sample SN-1. The low-magnification images show that the diameter of the NWs is ∼200 nm. The HRTEM image and FFT inset reveal a poor crystal with abundant stacking faults (SFs).

Another intriguing phenomenon is the formation of 1D nanostructures. As shown in figure 2, the average length of the NWs is estimated to be ∼8 *μ*m, accounting for a growth rate of ∼0.5 *μ*m min^−1^ (⩾1500 °C) for sample SN-1. PXRD patterns in figure 5(a) show evidence of *α*-SiC phase coexisting with the *α*/*β*-Si_3_N_4_ phases, which was further confirmed by Raman spectrum in figure 5(b). The peak at 784.7 cm^−1^ belongs to *α*-SiC, and its broadening shape indicates the disordered structure [28, 29]. Further investigations by TEM were made, as illustrated in figure 6. The average diameter of the nanowires is ∼200 nm. The EDX result shows that the composition of the body is SiC, which is consistent with the Raman indication. The high-resolution TEM (HRTEM) image shows the atomic resolution of the selected area of a nanowire. The fast fourier transform (FFT) pattern reveals low halo intensity with dots visible on sharp lines, indicating good order in two directions and low order in the third one. Such a poor crystal with abundant stacking faults is commonly seen in SiC NWs [30–34].

A large majority of studies have focused on the vapor–liquid–solid mechanism of SiC NWs in atmosphere pressure, and a few concern the case in vacuum. In our investigation, sintering of silicon nitride foams was performed in ‘vacuum’ (∼10 Pa after evacuation), and the formation mechanism of the SiC NWs was explored based on the thermodynamic calculations. To simplify, the assumption was made that the total equilibrium gas pressure during the entire formation process was fixed to 10 Pa. The possible reactions involved are listed as below:

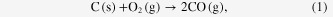


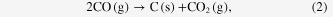


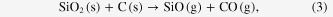


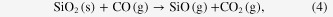


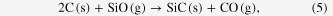


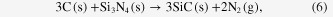


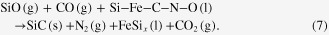



**Figure 7.**
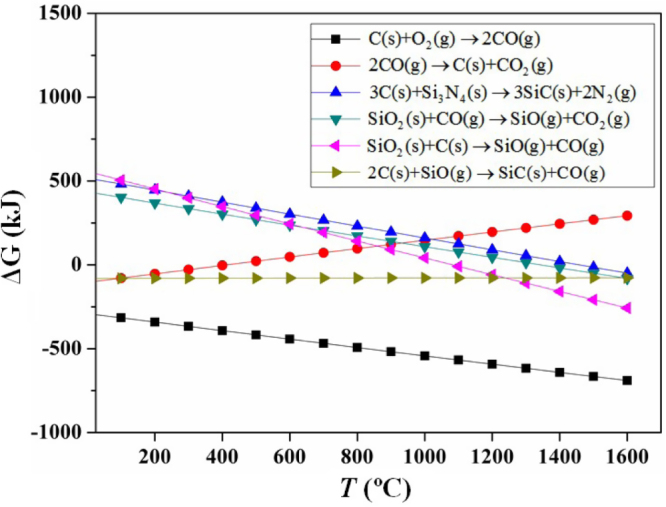
Thermodynamic calculations for the possible reactions occurring in the range of 25 ∼ 1600 °C assuming that the gas pressure during the entire process was 10 Pa at each equilibrium condition.

**Figure 8.**
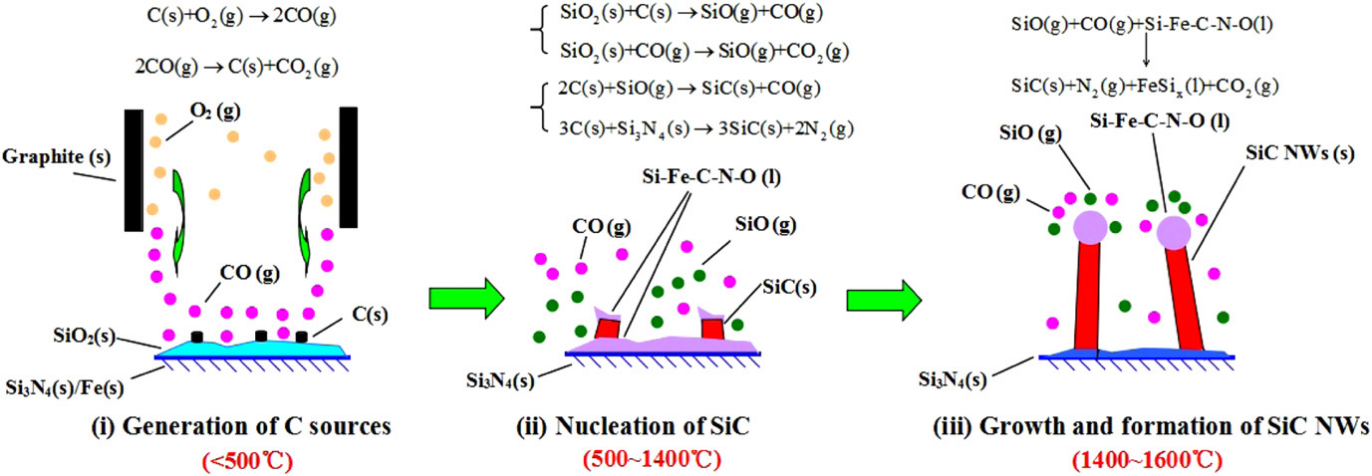
Schematic illustration for the vapor–liquid–solid formation mechanism of the SiC NWs.

The Gibbs free energy values were given for reactions (1) – (6) shown in figure 7 at 25 ∼ 1600 °C. As can be seen, the values become negative when the temperatures are < ∼500 °C for reaction (2), > ∼1000 °C for reactions (3), (4) and (6) and arbitrary for reactions (1) and (5). In this case, the possible formation process is proposed and illustrated in figure 8. Three steps were involved.

(i) Generation of carbon source (< 500 °C). The residual oxygen gas in the chamber reacted with graphite die/spacers and formed CO gas, which achieved an equilibrium with releasing carbon (solid) and CO_2_ [35, 36]. Previous investigations pointed out that only very low equilibrium oxygen partial pressures (∼10^−10^ Pa) are required to form CO gas [30, 31]. This pressure is lower than that from the oxygen impurity in the chamber.
(ii) Nucleation of SiC (500 ∼ 1400 °C). Silica layer that formed on the surface of Si_3_N_4_, interacted with solid state carbon and CO gas, generating SiO gas. Then the reactions between C, SiO and Si_3_N_4_ led to the formation of SiC nuclei (solid). At the same time C was dissolved into Si–Fe–N–O melt (liquid) and gave out liquid phase Si–Fe–C–N–O. Fe was from the raw powder and acted as a catalyst [5, 6].
(iii) Growth and formation of SiC NWs (1400 ∼ 1600 °C). SiO and CO vapors were constantly absorbed by the Si–Fe–C–N–O (liquid), followed by precipitation of SiC (solid) due to oversaturation. This enabled further growth of the NWs. The melt droplets remained attached to the tip after cooling, resulting in a semispherical cap.

Above all, the vapor–liquid–solid formation process of the SiC NWs under ‘vacuum’ is consistent with the typical tip-body structure observed from figures 2(b) and (c). This fast formation of NWs was supposed to be facilitated by the SITR process.

## 4. Conclusion

In summary, an intense thermal radiant sintering technique was developed to densify Si_3_N_4_ foams shaped by a surfactant-based direct foaming process, resulting in highly porous (∼80 vol%) Si_3_N_4_ foams possessing dense cell walls decorated with SiC NWs. Necking of grains occurred, and the resultant compressive strength was ∼10 MPa. The vapor–liquid–solid mechanism contributed to the formation of the nanostructures. The SITR process proved to be effective and can also be extended to consolidate other ceramic foams.
